# Ausbildung in und Anwendung von fokussierter Echokardiographie: Evaluierung des Ausbildungsstands und der klinischen Anwendung perioperativer fokussierter Echokardiographie durch AnästhesistInnen in Deutschland

**DOI:** 10.1007/s00101-026-01719-6

**Published:** 2026-08-13

**Authors:** Timo Kummerow, Pablo Cavalié, Felix Reinecke, Melissa Barroux, Matthias Göpfert, P. Scheiermann, R. Tomasi

**Affiliations:** Klinik für Anaesthesiologie, LMU Klinikum München, Marchioninistraße 15, 81377 München, Deutschland

**Keywords:** Fokussierter kardialer Ultraschall (FOCUS), Perioperatives Management, Anästhesiologie, Supervision, Fortbildung Echokardiographie, Focused cardiac ultrasound (FOCUS), Perioperative management, Anesthesiology, Supervision, Echocardiography continuing training

## Abstract

**Hintergrund:**

Der fokussierte kardiale Ultraschall (FOCUS) ist eine zielgerichtete Point-of-Care-Echokardiographie zur raschen Beurteilung kardialer Funktion und Morphologie und ist in Notfall- und Intensivmedizin etabliert.

**Fragestellung:**

Die Studie untersuchte Ausbildungsstand, Supervision und klinische Anwendung von FOCUS durch AnästhesistInnen in Deutschland sowie deren Zusammenhang mit der subjektiven Sicherheit bei der Durchführung von FOCUS.

**Studiendesign:**

Anonymisierte digitale Querschnittstudie unter ärztlichen DGAI-Mitgliedern mittels standardisiertem Fragebogen zu beruflichen Angaben, Kursausbildung, Supervision, klinischer Integration und Selbsteinschätzung praktischer FOCUS-Fertigkeiten.

**Ergebnisse:**

Insgesamt wurden 469 Rückmeldungen analysiert. Über 80 % hatten mindestens einen FOCUS-Basiskurs absolviert, mehr als 40 % berichteten jedoch keine Supervision vor eigenständiger Anwendung. Jeweils rund 40 % nutzten FOCUS regelmäßig bzw. unregelmäßig, 20 % gar nicht. Der häufigste Einsatz erfolgte im Umfeld von Notfällen oder auf der Intensivstation. Präoperativ wurden Echokardiographien bei elektiven PatientInnen überwiegend durch die Kardiologie (> 80 %), vor Notfalleingriffen häufiger auch durch AnästhesistInnen (> 60 %) durchgeführt. Die Kursausbildung war mit höherer Anwendungshäufigkeit und subjektiver Selbstsicherheit assoziiert; dieser Zusammenhang war für Supervision stärker.

**Diskussion:**

Trotz hoher Kursbeteiligung bleibt die perioperative Anwendung von FOCUS begrenzt. Kurse allein waren nur moderat mit Anwendungshäufigkeit und subjektiver Selbstsicherheit assoziiert. Für eine patientensichere Implementierung erscheinen daher kontinuierliche Anwendung, Supervision, sichere technische Durchführung, Kenntnis eigener Limitationen und strukturierte Ausbildungswege erforderlich.

## Abstract (en)

**Background:**

Focused cardiac ultrasound (FOCUS) is a targeted point-of-care echocardiographic approach for rapid assessment of cardiac function and morphology. It is established in emergency and intensive care medicine and increasingly more relevant for making perioperative decisions. Data on its integration into perioperative workflows by anesthesiologists and on the role of training and supervision, remain limited.

**Objective:**

This nationwide survey assessed FOCUS training, supervision and clinical application among anesthesiologists in Germany and examined how training and supervision were associated with self-reported confidence in performing FOCUS and the extent of its clinical use.

**Material and methods:**

We conducted an anonymized digital cross-sectional survey among physician members of the German Society of Anesthesiology and Intensive Care Medicine (DGAI). The standardized questionnaire comprised 21 items on professional background, echocardiographic training, supervised examinations, clinical integration of FOCUS and self-assessed skills. Training formats were grouped as no course, basic course, or basic plus advanced course. Supervision was recorded as the number of supervised FOCUS examinations after course attendance but the survey did not distinguish between bedside instruction, joint interpretation or image review. Complete datasets were analyzed using Pearson’s χ^2^-test and Wilson 95% confidence intervals.

**Results:**

Overall, 645 physicians participated and 469 completed the questionnaire. Senior physicians were overrepresented compared with the DGAI membership structure, whereas residents were underrepresented. More than 80% had completed at least one basic FOCUS course, yet more than 40% reported no supervised examinations before independently performing FOCUS in patients. Approximately 40% used FOCUS regularly, another 40% irregularly and 20% did not use it in practice. The FOCUS examination was used most often in emergency and intensive care situations, followed by intraoperative monitoring and preoperative evaluation. For elective preoperative assessment, cardiology was named most frequently, whereas before emergency surgery anesthesiologists were more often involved. Course participation was significantly associated with more frequent FOCUS use and supervised examinations showed an even stronger association with clinical use. Self-reported confidence in qualitative left ventricular function assessment and quantitative ejection fraction measurement increased with course level and supervision. Lack of qualification, insufficient supervision and time constraints were the main barriers.

**Conclusion:**

Despite high course participation and broad acceptance of FOCUS, the integration into perioperative practice remains limited. Courses alone appear insufficient for sustainable implementation. Supervision, continuous practice and structured longitudinal training pathways seem central to build confidence and support safe patient use. Beyond image interpretation, training should include correct device handling, suitable presets and exposure to normal and pathological findings. The use of FOCUS should be regarded as an adjunct to clinical assessment, not as a replacement for comprehensive cardiological echocardiography; it requires appropriate indications, technical proficiency and awareness of individual limitations. Interpretation is limited by the cross-sectional design, self-reported outcomes and selection bias towards a more experienced and FOCUS-affine subgroup.

## Zusammenfassung

Der fokussierte kardiale Ultraschall (FOCUS) gewinnt im perioperativen Management zunehmend an Bedeutung. In einer deutschlandweiten anonymisierten Online-Befragung unter AnästhesistInnen wurden Ausbildungsstand, Supervision und klinische Anwendung untersucht. Eine höhere Anwendungshäufigkeit von FOCUS war signifikant mit Kursausbildung und insbesondere mit einer größeren Zahl supervidierter Untersuchungen assoziiert. Die Ergebnisse betonen die Notwendigkeit strukturierter Ausbildungsprogramme für eine sichere klinische Implementierung.

## Hintergrund und Fragestellung

Als „Point-of-Care“-Echokardiographie dient FOCUS der gezielten Beurteilung kardialer Funktion und Morphologie bei akuten klinischen Fragestellungen. Je nach Situation und Expertise können qualitative und (semi)quantitative Parameter erhoben werden, sodass eine unmittelbare differentialdiagnostische Einschätzung kardiovaskulärer Pathologien möglich ist. [11, 15].

FOCUS ist in der Akutversorgung kritisch kranker PatientInnen, insbesondere in der Notfallmedizin und auf Intensivstationen, etabliert und gewinnt auch perioperativ an Bedeutung, da kardiovaskuläre Komplikationen zu den häufigsten Ursachen perioperativer Morbidität und Letalität zählen [6, 14]. Präoperativ ermöglicht FOCUS bei Elektiv- sowie bei Notfalleingriffen eine rasche Beurteilung der kardialen Funktion, sodass höhergradige Klappenvitien, Volumenmangel oder eine eingeschränkte Pumpfunktion frühzeitig erkannt und in die anästhesiologische Planung integriert werden können. Intraoperativ hilft FOCUS bei der Beurteilung hämodynamischer Veränderungen in Echtzeit.

FOCUS wird zunehmend auch von NichtkardiologInnen, insbesondere AnästhesistInnen, eigenständig durchgeführt. Es ersetzt die umfassende kardiologische transthorakale Echokardiographie (TTE) nicht, sondern ist als ergänzender Baustein der klinischen Gesamtbeurteilung zu verstehen – insbesondere bei zeitkritischen Entscheidungen und limitierten Ressourcen. Umfang und Fragestellungen wurden kürzlich in einer interdisziplinären S2k-Leitlinie definiert [11]. Studien belegen eine gute Übereinstimmung zwischen FOCUS-Befunden von NichtkardiologInnen und kardiologischen TTE [1, 5, 8, 16]. Daten dazu, welche Fachdisziplinen präoperative Echokardiographien im perioperativen Setting tatsächlich durchführen, fehlen bislang.

FOCUS-Kompetenzen werden in Deutschland vor allem über standardisierte Fortbildungscurricula der Deutschen Gesellschaft für Anästhesiologie und Intensivmedizin (DGAI) und der Deutschen Gesellschaft für Ultraschall in der Medizin (DEGUM) vermittelt. Grundkurse wie „Perioperative fokussierte Echokardiographie 1“ (PFE-1-Kurs) lehren Basisfertigkeiten, darunter die Einstellung standardisierter Schnittebenen sowie die morphologische Beurteilung kardialer Pathologien. Aufbaukurse (z. B. PFE-2-Kurs) vermitteln zusätzlich (semi-)quantitative Verfahren zur Beurteilung der ventrikulären Pumpfunktion und der Herzklappenfunktion. Diese Kursformate decken die zentralen Inhalte und praktischen Fertigkeiten für einen sicheren FOCUS-Einsatz im anästhesiologischen Alltag ab.

Neben der theoretisch-praktischen Ausbildung sind strukturierte Supervision und regelmäßige Anwendung entscheidend für eine sichere FOCUS-Nutzung. Unklar ist bislang, wie häufig erlernte Fertigkeiten tatsächlich eingesetzt werden, und welchen Beitrag Kurse und Supervision zur nachhaltigen Integration leisten. Daher wurden in einer deutschlandweiten Befragung unter ärztlichen DGAI-Mitgliedern die aktuelle Ausbildungssituation, klinische Integration und subjektive Handlungssicherheit in Abhängigkeit von Ausbildung und Supervision untersucht. Die Ergebnisse sollen dazu beitragen, zukünftige Ausbildungsstrategien zu optimieren und die Implementierung von FOCUS im anästhesiologischen Alltag zu stärken.

## Studiendesign

Die vorliegende Untersuchung ist eine anonymisierte, digitale Querschnittstudie zur Erfassung von Ausbildung und Einsatz von FOCUS in der Anästhesiologie. Primäres Ziel war die Darstellung des aktuellen Qualifikationsstandes sowie der Integration von FOCUS in perioperative Abläufe. Sekundär wurden die subjektive Selbsteinschätzung von FOCUS-Fähigkeiten in Abhängigkeit von der echokardiographischen Ausbildung erhoben.

Die Studie wurde von der Ethikkommission der Ludwig-Maximilians-Universität München genehmigt (Projekt Nr. 22-0821) und von der Klinik für Anaesthesiologie des LMU-Klinikums in Kooperation mit dem Wissenschaftlichen Arbeitskreis Ultraschall der DGAI durchgeführt. Eingeladen wurden AnästhesistInnen aller Weiterbildungsstufen über den zentralen E‑Mail-Verteiler der DGAI. Nur nahezu vollständige Datensätze mit maximal einer fehlenden Einzelangabe wurden ausgewertet. Fehlende Werte betrafen Supervision (*n* = 10), Beurteilung der linksventrikulären Pumpfunktion (*n* = 5) und berufliche Position (*n* = 2). Die Teilnahme war freiwillig; eine numerische Pseudonymisierung stellte die Anonymität sicher und schloss eine Rückverfolgbarkeit auf Personen oder Einrichtungen aus.

Der digitale Fragebogen wurde mit der datenschutzkonformen Software LimeSurvey (Version 6.5) erstellt und war vom 10. Juni bis zum 8. Juli 2024 über einen zugesandten Link abrufbar. Die Inhalte wurden in Zusammenarbeit mit dem Wissenschaftlichen Arbeitskreis Ultraschall der DGAI entwickelt.

Der Fragebogen umfasste 21 Punkte zu vier Abschnitten:

1. berufliche Angaben (Weiterbildungsstand, Einsatzbereich, Klinikgröße)
2. Ausbildung in (fokussierter) transthorakaler Echokardiographie
3. klinische Integration von FOCUS im perioperativen Alltag
4. Selbsteinschätzung praktischer FOCUS-Fähigkeiten

Die Abschnitte 1–3 bestanden aus Multiple-Choice-Fragen mit Einfach- und Mehrfachantworten sowie optionalen Freitextfeldern. Im Abschnitt 4 erfolgte die Selbsteinschätzung spezifischer Untersuchungsschritte mittels numerischer Likert-Skalen zur Bewertung von subjektiver Selbstsicherheit und Supervisionsbedarf. Die erfassten Kursformate der DGAI, DEGUM und weiterer Anbieter wurden nachträglich in drei Kategorien gruppiert: „Basiskurs“, „Aufbaukurs“ und „Kein Kurs“. Zu den Basiskursen zählten PFE 1 der DGAI, der „Basiskurs Notfallsonographie“ sowie der „Grundkurs II Anästhesie“ der DEGUM. Diese Kurse umfassten typischerweise etwa 10 Unterrichtseinheiten (UE ≈ eine Zeitstunde) mit rund 50 %igem Praxisanteil und vermittelten standardisierte Schallebenen sowie die morphologische Beurteilung kardialer Pathologien. Aufbaukurse umfassten unter anderem den PFE 2‑Kurs der DGAI sowie den Grund- und Aufbaukurs Dopplerechokardiographie der DEGUM. Aufbaukurse beinhalteten mindestens 8–20 UE über einen bis vier Kurstage, ebenfalls mit ca. 50 % praktischen Anteilen. Vermittelt wurden erweiterte Schallebenen und (semi)quantitative Verfahren zur Beurteilung der ventrikulären Funktion und der Herzklappen, teils an Patienten mit pathologischen Befunden (PFE 2‑Kurs DGAI). Kurse mit primärem Fokus auf Peri-arrest- bzw. Reanimationssituationen wurden der Kategorie „Kein Kurs“ zugeordnet, da sie nicht das Spektrum standardisierter transthorakaler Schnittebenen und kardialer Funktions- bzw. Klappenbeurteilung abbilden.

Supervision wurde als Anzahl supervidierter FOCUS-Untersuchungen nach Kursbesuch erfasst; zwischen direkter Anleitung, gemeinsamer Befundung und nachträglicher Bild‑/Befundbesprechung wurde nicht unterschieden. Die Datenaufbereitung erfolgte in Microsoft Excel, die statistische Analyse in R (Version 4.5.1). *p*-Werte wurden mittels Pearson-Chi^2^-Test berechnet (df abhängig von der Anzahl der Vergleichsgruppen). 95 %-Konfidenzintervalle wurden nach Wilson bestimmt. Es wurde ein zweiseitiges Signifikanzniveau von α = 0,05 verwendet; *p* < 0,05 galt als statistisch signifikant, *p* < 0,001 als hochsignifikant.

## Ergebnisse

Insgesamt gingen 645 Rückmeldungen ein; 469 nahezu vollständige Datensätze wurden analysiert (Rücklaufquote ca. 4,4 %). Die Auswertung erfolgte mit variablenspezifischen Nennern. Die Verteilung der Berufsgruppen, FOCUS-Anwendung, Ausbildung und Supervision ist in Tab. 1 dargestellt. Im Vergleich zur DGAI-Mitgliederstruktur waren Ober- und ChefärztInnen überrepräsentiert, AssistenzärztInnen hingegen unterrepräsentiert. Mit zunehmender klinischer Position zeigten sich eine häufigere regelmäßige FOCUS-Anwendung sowie höhere Anteile an Basis- und Aufbaukursen und umfangreicherer Supervision.

**Tab. 1:** Verteilung von beruflicher Position, FOCUS-Anwendung, Kursausbildung und Supervision innerhalb der Studienpopulation im Vergleich zur DGAI-Mitgliederstruktur.

	Ausbildungsstand (Anteil)		Anwendungshäufigkeit von FOCUS (in %)			Ausbildung in FOCUS (Kurse) (in %)			Ausbildung in FOCUS (Anzahl der Supervisionen) (in %)			
	Studienpopulation	DGAI-Mitgliedsstruktur	Regelmäßig	Unregelmäßig	Nie	Kein Kurs	Basiskurs	Basis- und Aufbaukurs	> 20	10–20	< 10	Keine
AssistenzärztInnen	15,4 %	38,7 %	19,4	48,6	31,9	38,9	33,3	27,8	7,1	10,0	21,4	60,0
FachärztInnen	22,3 %	26,8 %	35,6	35,6	28,8	24,0	29,8	46,2	12,6	18,4	22,3	46,6
OberärztInnen	47,8 %	27,1 %	43,5	44,4	12,1	11,6	35,4	52,9	24,2	20,6	16,1	39,0
ChefärztInnen	14,6 %	7,2 %	55,2	31,3	13,4	17,6	22,1	60,3	36,9	18,5	6,2	38,5
*Insgesamt*	*n=467*	*n=10.591*	*39,7*	*41,2*	*19,1*	*19,5*	*31,9*	*48,6*	*20,8*	*20,4*	*16,9*	*43,8*

„Regelmäßig“ täglich bis mehrmals wöchentlich, „unregelmäßig“ alle ein bis 8 Wochen. Angaben in Prozent in Bezug auf die jeweilige Berufsgruppe. Für die nach beruflicher Position stratifizierte Analyse lagen 466 vollständige Angaben vor

**Abb. 1:**
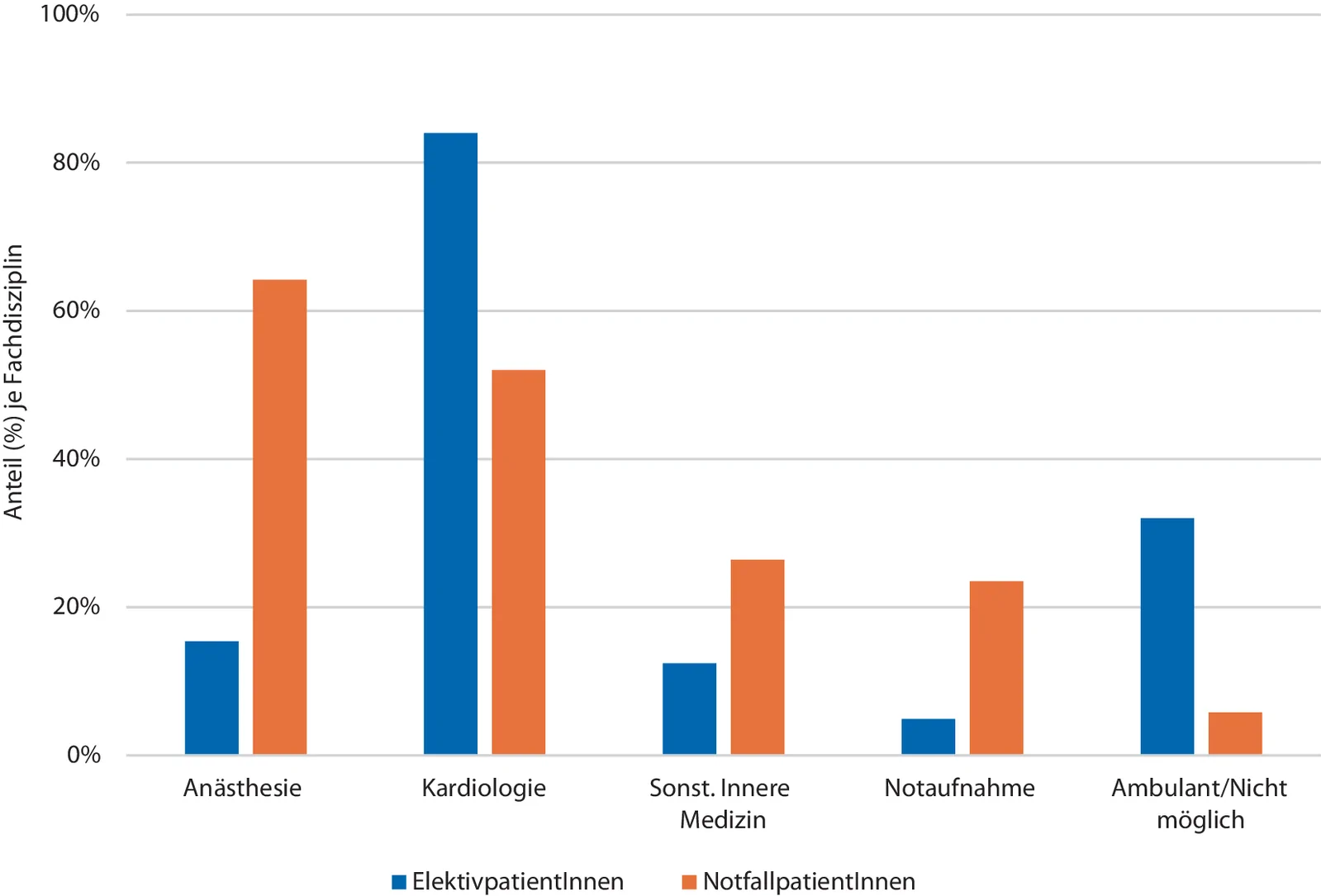
Häufigkeit der Nennung von Fachabteilungen, die präoperative TTE- oder FOCUS-Untersuchungen bei Elektiv- und NotfallpatientInnen durchführen (*n* = 469; Mehrfachnennungen möglich)

FOCUS wurde überwiegend in Notfallsituationen und bei intensivmedizinischen Entscheidungen eingesetzt (85,2 %), gefolgt vom intraoperativen Monitoring (52,9 %) und der präoperativen Evaluation (42,2 %) Für die präoperative Evaluation wurde zudem erhoben, welche Fachdisziplinen TTE- bzw. FOCUS-Untersuchungen bei elektiven und notfallmäßigen PatientInnen durchführen (Abb. 1).

**Abb. 2:**
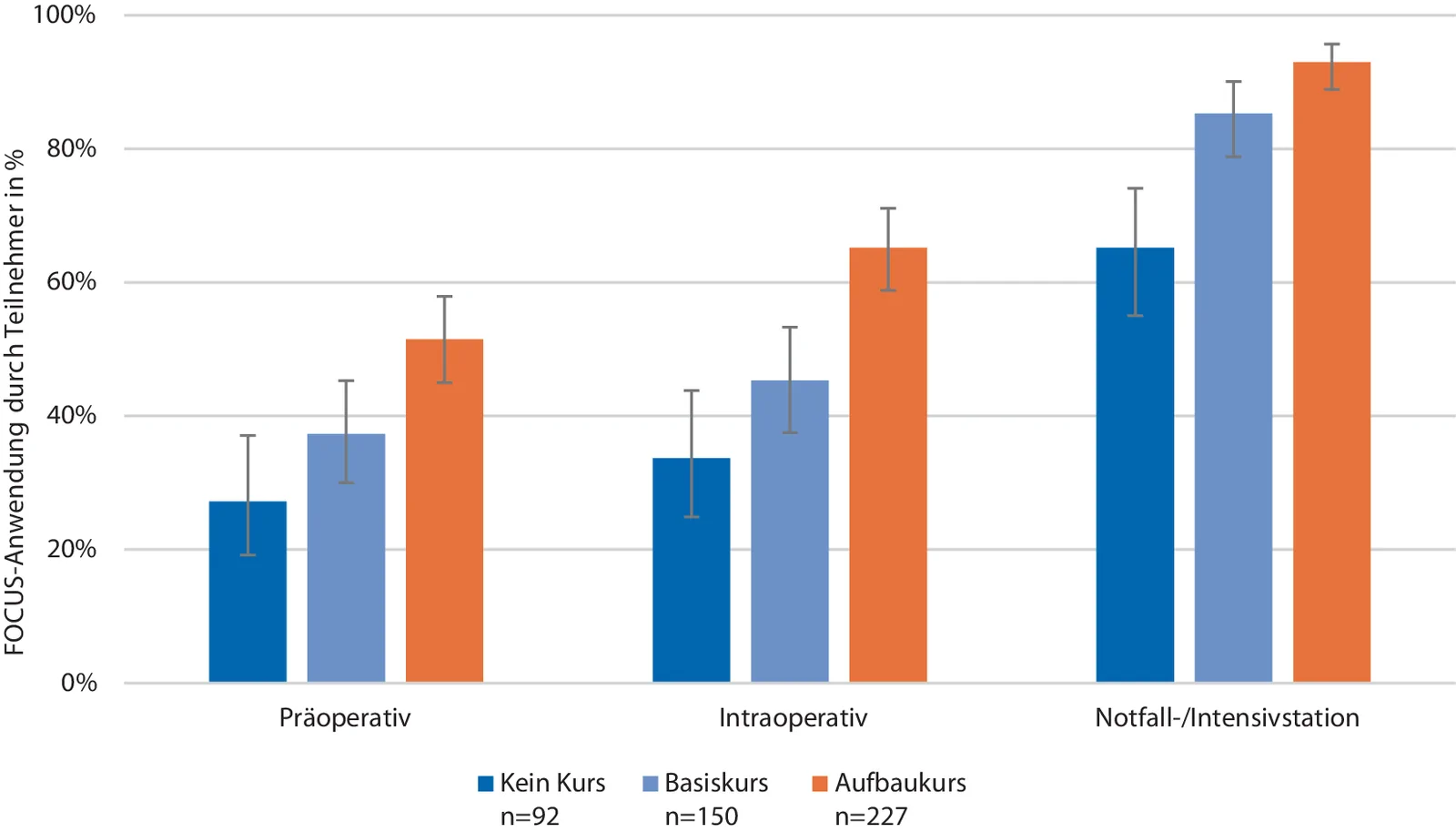
FOCUS-Anwendungsraten nach Kursniveau. *N* = 469. 95 %-Konfidenzintervall (Wilson). Zwischen den Gruppen zeigten sich in allen Bereichen signifikante Unterschiede (Chi^2^, *p* < 0,001)

Der Besuch eines Basis- oder Aufbaukurses war mit einer signifikant häufigeren Durchführung von FOCUS-Untersuchungen in Notfallsituationen, auf der Intensivstation und im perioperativen Setting assoziiert (Abb. 2).

Aufgrund der hohen Zahl an FOCUS-Anwendungen ohne absolvierten Basis- oder Aufbaukurs wurden zusätzlich die Angaben zur Supervision analysiert. Von den Teilnehmenden ohne Kursteilnahme und ohne Supervision setzten 63 % FOCUS in Notfallsituationen ein (38/60); für prä- bzw. intraoperative Anwendungen betrugen die entsprechenden Anteile ebenfalls 60 % (15/25 bzw. 19/31). Auch unter AbsolventInnen von Basis- bzw. Aufbaukursen führten 38 % (49/128) bzw. 26 % (54/211) FOCUS in Notfallsituationen ohne vorherige Supervision durch. Prä- oder intraoperative FOCUS-Anwendungen wurden in dieser Gruppe von etwa einem Drittel bzw. einem Viertel der Teilnehmenden ohne supervidierte Untersuchungen berichtet.

**Abb. 3:**
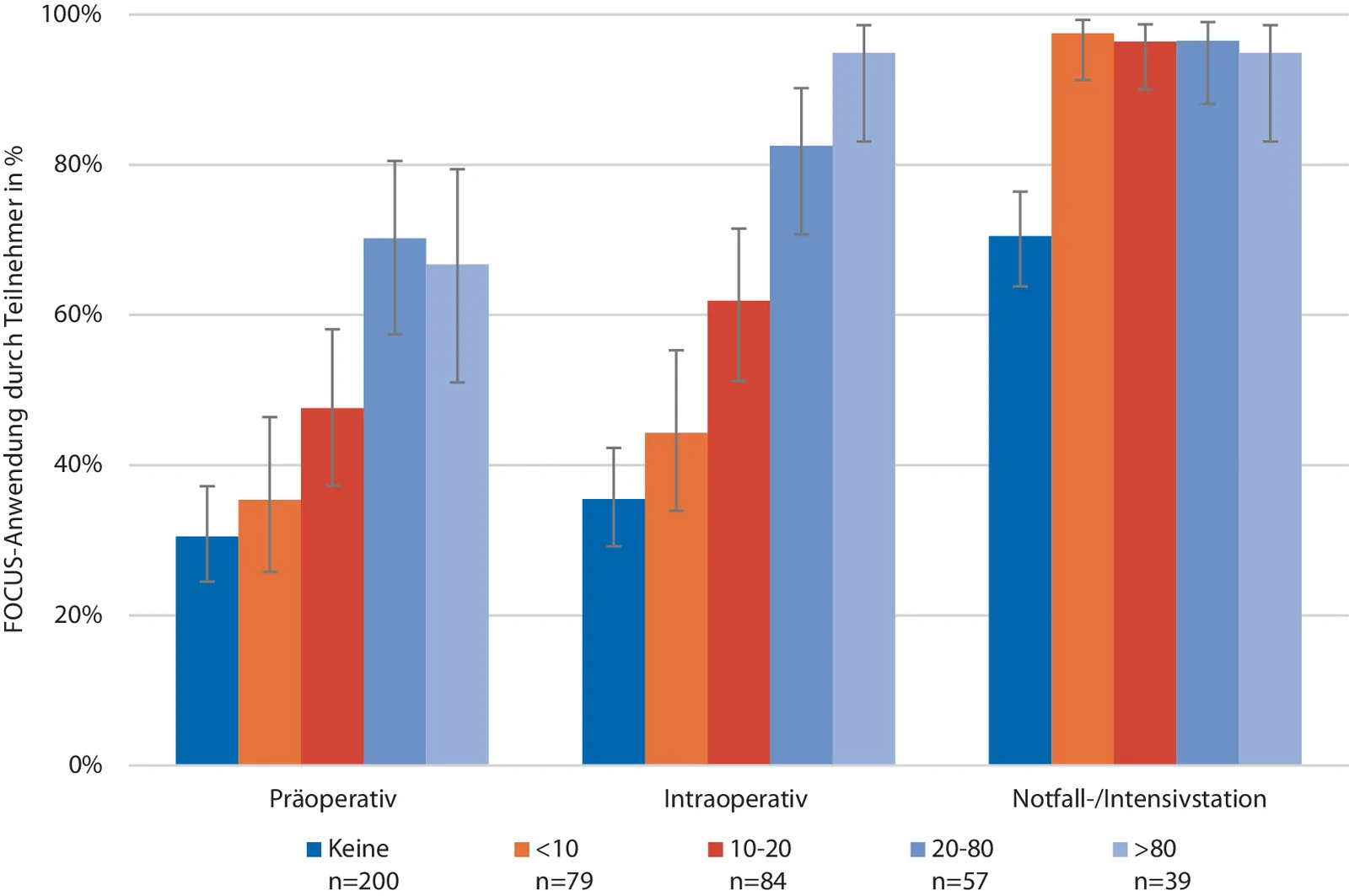
FOCUS-Anwendungsraten nach Supervision. *N* = 459. 95 %-Konfidenzintervall (Wilson). Zwischen den Gruppen zeigten sich in allen Bereichen signifikante Unterschiede (Chi^2^, *p* < 0,001)

Insgesamt war eine höhere Zahl supervidierter Untersuchungen mit einer häufigeren klinischen Nutzung von FOCUS assoziiert (Abb. 3).

Sowohl Kursniveau als auch Supervision waren mit dem klinischen FOCUS-Einsatz assoziiert, wobei Supervision eine stärkere Assoziation zeigte.

**Abb. 4:**
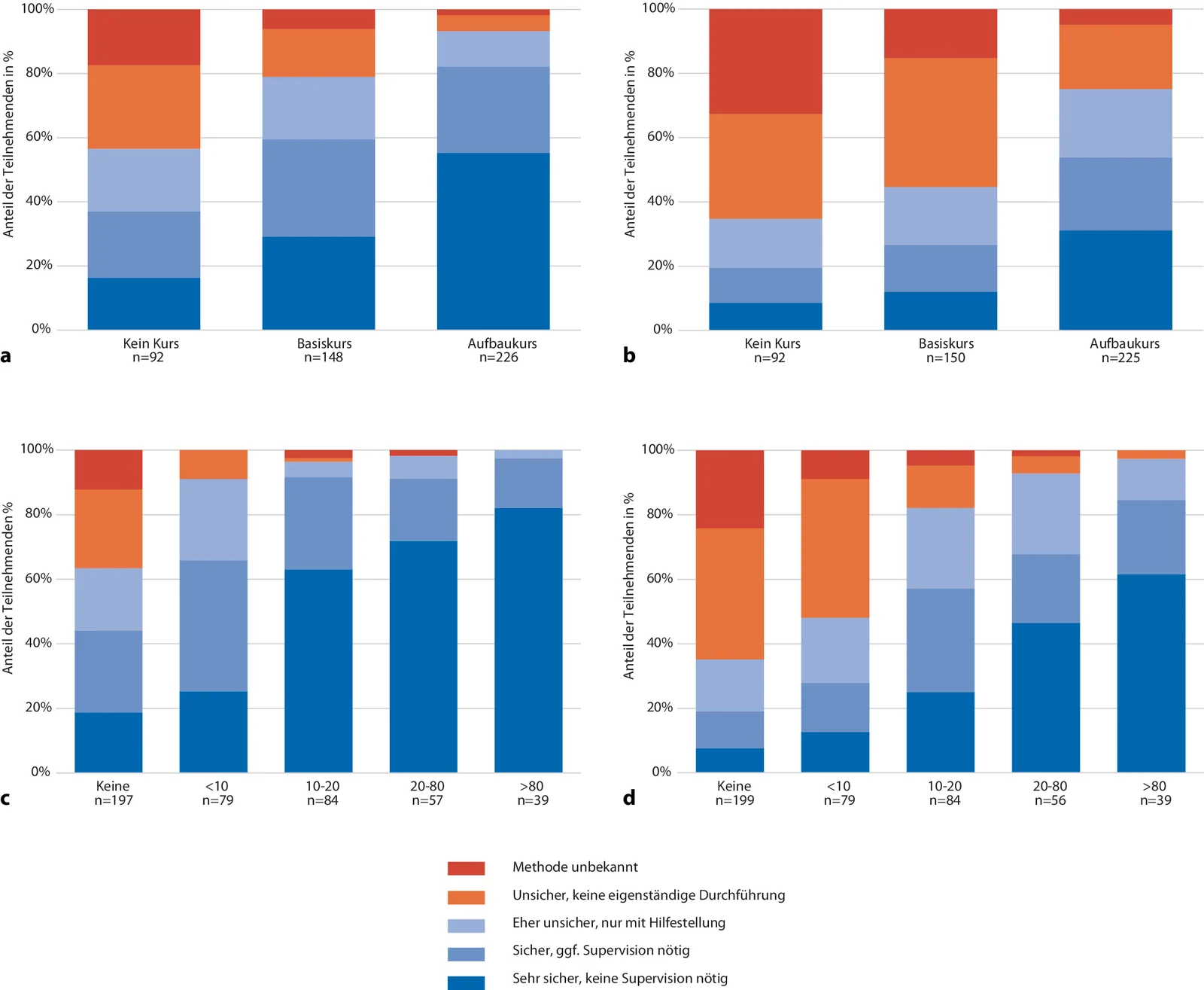
Einschätzung der Selbstsicherheit **a***„Eyeballing“* der Ejektionsfraktion in Abhängigkeit vom Kursniveau (*n* = 467). Unterschied zwischen den Gruppen signifikant (X2 = 89,5; df = 8; *p* < 0,001). **b***Messung* der Ejektionsfraktion (z. B. Simpson-Methode) in Abhängigkeit vom Kursniveau (*n* = 467). Unterschied zwischen den Gruppen signifikant (X2 = 81,95; df = 8; *p* < 0,001). **c***„Eyeballing“* der Ejektionsfraktion in Abhängigkeit von der Anzahl durchgeführter Supervisionen (*n* = 456). Unterschied zwischen den Gruppen signifikant (X2160,94; df = 16; *p* < 0,0001). **d** (*n* = 457). **d***Messung* der Ejektionsfraktion (z. B. Simpsons-Methode) nach Anzahl der Supervisionen, Unterschied zwischen den Gruppen signifikant (X2165,2; df = 16; *p* < 0,001)

Über alle Kursstufen hinweg war eine höhere Supervision (> 10 Untersuchungen) mit deutlich höheren Anwendungsraten verbunden: Präoperativ stieg die Nutzung ohne Kurs von 24 % (geringe Supervision) auf 46 % (hohe Supervision). Ähnliche Effekte zeigten sich bei Basis- und Aufbaukursen (Basiskurs 32 % vs. 51 %, Aufbaukurs 38 % vs. 63 %). Der Kursbesuch allein war nur mit moderaten Unterschieden verbunden, während intensivere Supervision konsistent mit einer stärkeren Integration von FOCUS in den klinischen Alltag einherging (Daten nicht graphisch dargestellt). Zur Beurteilung der Selbstsicherheit wurden zwei zentrale Fertigkeiten analysiert: die subjektive Einschätzung der linksventrikulären Pumpfunktion („Eyeballing“) und deren quantitative Messung (z. B. Simpsons-Methode). Die Teilnehmenden wurden nach Kursniveau und Zahl supervidierter Untersuchungen kategorisiert. Die Selbstsicherheit korrelierte sowohl mit dem Kursniveau als auch mit der Zahl supervidierter Untersuchungen (Abb. 4a–d). Ein ähnlicher Trend fand sich für weitere im Curriculum abgefragte TTE-/FOCUS-Fertigkeiten, etwa die morphologische und semiquantitative Klappenbeurteilung (Daten nicht dargestellt).

91,5 % der Befragten stimmten zu, dass FOCUS die perioperative Patientenversorgung verbessert (70,4 % volle, 21,1 % eher Zustimmung); 6,4 % äußerten sich neutral, 1,9 % lehnten die Aussage ab.

Als wichtigste Hindernisse im klinischen Alltag nannten die Teilnehmenden fehlende persönliche Qualifikation (60 %), unzureichende Supervision (59 %) und Zeitmangel (56 %). Eine eingeschränkte Geräteverfügbarkeit wurde deutlich seltener als Barriere genannt (33 %).

## Diskussion

Die Umfrage unterstreicht den Stellenwert von FOCUS in der klinischen Entscheidungsfindung und das Bestreben vieler AnästhesistInnen, ihre diagnostischen Kompetenzen zur Verbesserung der perioperativen Betreuung und Patientensicherheit zu erweitern. Trotz hoher Kursteilnahme mit mindestens einem absolvierten Basiskurs bei 80 % der Befragten wurde FOCUS von 60 % nicht oder nur unregelmäßig eingesetzt. Dies spricht für eine unzureichende klinische Integration trotz vorhandener Ausbildung.

FOCUS wird überwiegend reaktiv in Notfallsituationen eingesetzt, während prä- und intraoperative Anwendungen selten bleiben. Dies spricht für ein bislang nichtausgeschöpftes perioperatives Anwendungspotenzial. FOCUS ist dabei als ergänzender Baustein der klinischen Basisdiagnostik zu verstehen, ersetzt keine umfassende kardiologische Diagnostik und erfordert eine sorgfältige Indikationsstellung sowie die Kenntnis eigener Limitationen.

Auf Intensivstationen wird FOCUS aufgrund der akuten klinischen Dringlichkeit und der unmittelbaren therapeutischen Konsequenzen niedrigschwellig eingesetzt [9]. Präoperativ übernehmen AnästhesistInnen Echokardiographien aufgrund fehlender Qualifikation, begrenzter Supervision und Zeitmangels nur selten selbst; die Untersuchungen erfolgen überwiegend durch die Kardiologie. Ein strukturierter präoperativer Einsatz durch AnästhesistInnen könnte jedoch dazu beitragen, RisikopatientInnen frühzeitig zu identifizieren und kardiologische Ressourcen gezielter einzusetzen. Der Nutzen eines solchen Screeningansatzes ist bislang wenig bzw. nur in anderen klinischen Situationen (z. B. SchlaganfallpatientInnen [4]) untersucht. Entsprechende perioperative Daten werden derzeit in einer prospektiven Studie am Klinikum der Autoren erhoben [Projekt Nr.: 22-0821].

Eine Metaanalyse zeigte, dass die perioperative Echokardiographie häufig zu diagnostischen und therapeutischen Anpassungen führt (> 50 %) [9]. Ein Einfluss des präoperativen FOCUS auf harte klinische Endpunkte ist jedoch bislang nicht belegt, auch nicht in randomisierten Studien wie ECHONOF II und PREOPFOCUS [2, 17].

Für die präoperative Risikostratifizierung sind vor allem die linksventrikuläre Funktion, höhergradige Aorten- und Mitralklappenvitien sowie Zeichen einer rechtsventrikulären Belastung relevant [3, 7, 12,13]. Mittel- bis hochgradige Pathologien lassen sich auch fokussiert meist zuverlässig erkennen. Studien zeigen für das „Eyeballing“ der linksventrikulären Funktion sowie die Einschätzung von Aortenstenosen eine gute Übereinstimmung zwischen unerfahrenen Untersuchenden und KardiologInnen [1, 5, 8, 16]. Grundlegende Messverfahren sind somit mit begrenzten Ressourcen erlernbar, während komplexe Fragestellungen – etwa Low-Flow-Low-Gradient-Aortenstenosen oder die diastolische Dysfunktion – deutlich mehr Expertise erfordern [19].

Der klinische Einsatz von FOCUS wurde sowohl durch Kursniveau als auch durch Supervision beeinflusst, wobei praktische Supervision die deutlich stärkere Assoziation zeigte. ÄrztInnen mit mehr Supervision nutzten FOCUS kursunabhängig signifikant häufiger, während der Kursbesuch allein nur mit moderaten Unterschieden verbunden war. Gleichzeitig erscheint die Supervision nach dem Kursabschluss unzureichend: Über 60 % der Befragten gaben an, keine oder weniger als zehn supervidierte Untersuchungen durchgeführt zu haben.

Vor dem Hintergrund des „Train-to-maintain“-Prinzips stellt sich die Frage, wie viele Untersuchungen und wie viel Supervision erforderlich sind, um FOCUS-Fertigkeiten zu erlernen und langfristig zu erhalten. Internationale Empfehlungen, u. a. der American Society of Echocardiography (ASE), nennen hierfür Mindestzahlen supervidierter Untersuchungen [10]; vergleichbare Anforderungen finden sich im DGAI-Zertifikat „Perioperative fokussierte Echokardiographie (PFE)“ [18]. Diese Anforderungen stellen jedoch keine Schwelle für eine sichere diagnostische Kompetenz dar, die in der Regel eine darüber hinausgehende, kontinuierliche Anwendung und Supervision erfordert. Fehlinterpretationen können im perioperativen Kontext unmittelbare therapeutische Konsequenzen haben; neben der Detektion pathologischer Befunde ist daher die sichere Identifikation von Normalbefunden essenziell. Eine ergänzende klinische Exposition, beispielsweise im Rahmen von Hospitationen, erscheint hierfür sinnvoll. Eine zusätzliche Voraussetzung für valide Befunde ist die korrekte technische Durchführung der Untersuchung. Geräteeinstellungen und geeignete Presets beeinflussen die Bildqualität und damit die Interpretation erheblich und sollten daher als integraler Bestandteil der Ausbildung verstanden werden.

Die subjektive Selbstsicherheit korrelierte mit dem Kursniveau und der Supervision. Basiskurse waren für einfache morphologische Beurteilungen meist ausreichend; etwa zwei Drittel der Teilnehmenden fühlten sich hierbei sicher. Für komplexere Verfahren waren hingegen zusätzliche Ausbildung und Supervision erforderlich, um eine vergleichbare Selbstsicherheit zu erreichen (Abb. 4).

Unsere Umfrage deutet auf eine deutliche Diskrepanz zwischen der angestrebten Ausbildungsstruktur – Kursbesuch, anschließende Supervision und strukturierte Zertifizierung – und der tatsächlichen klinischen Umsetzung hin.

Die European Association of Cardiovascular Imaging (EACVI) beschreibt FOCUS als strukturierte, longitudinale Weiterbildung mit initialem Kurs, anschließender Supervision, eigenständig durchgeführten und nachbesprochenen Untersuchungen sowie dokumentierter Untersuchungserfahrung im Logbuch mit normalen und definierten pathologischen Befunden [15]. Eine solche Ausbildung erscheint essenziell für eine patientensichere Anwendung. Zertifikate können Kompetenzen formalisieren, sind jedoch mit erheblichem zeitlichem und personellem Aufwand verbunden. Für eine nachhaltige Implementierung von FOCUS im perioperativen Setting sollten daher neben Kursen auch klinische Hospitationen, klinikinterne SupervisorInnen und standardisierte Logbücher stärker berücksichtigt werden.

## Limitationen

Die heterogene Teilnehmendenstruktur, von AssistenzärztInnen bis zu erfahrenen KardioanästhesistInnen, kann die Vergleichbarkeit einschränken. Die Kompetenzangaben beruhen auf Selbsteinschätzungen und bilden daher subjektive Sicherheit, nicht jedoch objektive diagnostische Leistungsfähigkeit ab. Über- oder Unterschätzungen sind möglich; eine Validierung wäre nur durch den direkten Vergleich mit kardiologischen TTE-Befunden möglich.

AssistenzärztInnen waren in der Stichprobe unterrepräsentiert, Chef- und OberärztInnen hingegen überrepräsentiert. Da erfahrenere ÄrztInnen möglicherweise häufiger Zugang zu FOCUS, größere Anwendungserfahrung und höhere Selbstsicherheit besitzen, könnten Anwendung und subjektive Kompetenz überschätzt worden sein. Die Ergebnisse spiegeln daher wahrscheinlich eher eine erfahrene und FOCUS-affine Subgruppe als die gesamte anästhesiologische Ärzteschaft wider. Die freiwillige Teilnahme über die DGAI könnte diese Selektionsverzerrung verstärkt haben. Während die Basiskurse hinsichtlich des Inhalts und der Dauer weitgehend homogen waren, unterschieden sich die Aufbaukurse teils deutlich; zudem wurde Supervision nicht nach direkter Anleitung, gemeinsamer Befundung oder nachträglicher Bild‑/Befundbesprechung differenziert. Trotz moderater, aber früheren DGAI-Erhebungen vergleichbarer Rücklaufquote bietet die Studie eine aktuelle Momentaufnahme und wichtige Hinweise zu Einstellungen und Trends innerhalb der Fachgesellschaft.

## Fazit für die Praxis

- Hohe Akzeptanz und großes Interesse an FOCUS, jedoch bislang nur begrenzte Integration in den klinischen Alltag.
- Anwendung erfolgt überwiegend in Notfallsituationen; prä- und intraoperative Untersuchungen durch AnästhesistInnen bleiben selten.
- Ein relevanter Anteil nutzt FOCUS ohne Supervision und/oder formale Kurse.
- Der klinische Einsatz war stärker mit praktischer Supervision als mit dem reinen Kursbesuch assoziiert.
- Der Einfluss von FOCUS auf patientenrelevante Endpunkte wie Mortalität und Komplikationen wird derzeit in Studien untersucht.
- Eine strukturierte, longitudinal angelegte Ausbildung nach dem EACVI-Vorbild könnte die Implementierung verbessern und langfristig die Versorgungsqualität erhöhen.

## Data Availability

Die erhobenen Datensätze können auf begründete Anfrage in anonymisierter Form beim korrespondierenden Autor angefordert werden. Die Daten befinden sich auf einem Datenspeicher der Klinik für Anaesthesiologie des LMU Klinikums München.
